# Tumor‐derived exosomal miRNA‐320d as a biomarker for metastatic colorectal cancer

**DOI:** 10.1002/jcla.23004

**Published:** 2019-08-16

**Authors:** Youyong Tang, Yajing Zhao, Xingguo Song, Xianrang Song, Limin Niu, Li Xie

**Affiliations:** ^1^ School of Medicine and Life Sciences University of Jinan, Shandong Academy of Medical Sciences Jinan China; ^2^ Department of Clinical Laboratory, Shandong Cancer Hospital and Institute Shandong First Medical University and Shandong Academy of Medical Sciences Jinan China; ^3^ Shandong Provincial Key Laboratory of Radiation Oncology, Shandong Cancer Hospital and Institute Shandong First Medical University and Shandong Academy of Medical Sciences Jinan China

**Keywords:** biomarker, colorectal cancer, exosomes, metastasis, miR‐320d

## Abstract

**Background:**

To identify specific exosomal microRNAs (miRNAs) as serum biomarkers for prediction of metastasis in patients with colorectal cancer (CRC).

**Materials and Methods:**

Serum exosomes were isolated from patients with metastatic CRC (n = 34) and non‐metastatic CRC (n = 108) by ultracentrifugation and characterized using transmission electron microscopy, qNano, and Western blot. Differential exosomal miRNAs were screened by sequencing and validated by qPCR in metastatic and non‐metastatic CRC patients.

**Results:**

After sequence analysis, KEGG analysis showed that differential genes were associated with Rap1 signaling pathway and pathways in cancer, 6 upregulated exosomal miRNAs (miR‐224‐5p, miR‐548d‐5p, miR‐200a‐3p, miR‐320d, miR‐200b‐3p, and miR‐1246), and 3 downregulated exosomal miRNAs (novel_246, novel_301, and miR‐27a‐5p) were screened with fold change >1.5, among which miR‐320d was selected as the best candidate involved in CRC metastasis. Validation analysis revealed exosomal miR‐320d could significantly distinguish metastatic from non‐metastatic CRC patients (*P* = .019), with AUC of 0.633 for the diagnosis of patients with metastatic CRC. Besides, the combination of miR‐320d and CEA had an area under curve (AUC) of 0.804 for the diagnosis of patients with metastatic CRC.

**Conclusion:**

Serum exosomal miR‐320d is a promising non‐invasive diagnostic biomarker for distinguishing metastatic from non‐metastatic CRC.

## 1 INTRODUCTION

Colorectal cancer (CRC) is the third most prevalent cancer and the fourth most common cause of cancer‐related deaths worldwide, with around 1.2 million new diagnoses and more than 600 000 deaths every year.^1^ Poor prognosis and survival rate are mainly due to metastasis.^2^ Furthermore, approximately 50%‐60% of patients with CRC develop metastatic CRC (mCRC), and 80%‐90% have unresectable liver metastases^3^; thereby, it is critical to identify novel and highly sensitive CRC‐specific biomarkers for mCRC diagnostics.

Exosomes are nanoscale vesicles (40‐100 nm) derived from the luminal membranes of multivesicular bodies that are released by fusion with the cell membrane.^4^ Recent evidence indicated that exosomes act as a diagnostic biomarker in lung cancer,^5^ ovarian cancer,^6^ and pancreatic cancer.^7^ Exosomes have been implicated in the tumor metastatic process to normal tissues via the transfer of cancer‐specific cargo (ie, microRNA, RNAs and proteins),^8^ one of which the most exciting molecular marker for tumor diagnosis is microRNA (miRNA).^9^

MiRNAs are short (20‐24 nt) non‐coding RNAs that are involved in the post‐transcriptional regulation of gene expression in multicellular organisms by affecting both the stability and translation of mRNAs.^9^ Furthermore, numerous studies indicated that exosomes contain high levels of miRNAs, which have been shown to contribute to immunomodulation, chemo‐resistance, and metastasis in multiple tumor types.^10^ More significantly, circulating exosome‐encapsulated miRNAs have been demonstrated to play crucial roles in colorectal carcinogenesis as well as the prognosis of patients with CRC.^11^, ^12^, ^13^ Therefore, exosomal miRNA levels in the serum of cancer patients could be a diagnostic biomarker for metastasis.

In this study, we utilized RNA‐sequence analysis and quantitative PCR to analyze exosomal miRNAs from serum of CRC patients. We analyzed miRNA profiles in circulating exosomes and examined whether exosomal miRNA expression levels had a diagnostic value for mCRC patients.

## 2 MATERIALS AND METHODS

### 2.1 Patients

A total of 142 patients diagnosed with CRC were enrolled in this study at the Shandong Cancer Hospital between January 2018 and July 2018. Written informed consent was obtained from all participants. Tumor staging was estimated according to AJCC Cancer Staging Handbook of the American Joint Committee on Cancer, 2010. The patients did not receive any anti‐tumor treatment before peripheral blood collection or suffer from any other endocrine, immune, or metabolic diseases. Patient characteristics and history of diabetes are shown in Table 1.

**Table 1:** Characteristics of CRC patients for differentially expressed exosomal miR‐320d

Characteristics		No. cases	Median with interquartile range	*P*‐value
Age (y)	＜61	69	3.3800 (2.8021‐3.5356)	.925
	≥61	73	2.9800 (2.6996‐3.5206)	
Gender	Male	95	3.3750 (2.9125‐3.6141)	.061
	Female	47	2.9000 (2.4447‐3.3051)	
Drinking status	Yes	29	3.0150 (2.2656‐3.4189)	.232
	No	113	3.2050 (2.9026‐3.5268)	
Diabetes status	No	126	3.1350 (2.9218‐3.4566)	.601
	Yes	16	2.8725 (1.4275‐4.0537)	
Tumor position	Rectum	90	3.1475 (2.7402‐3.4795)	.663
	Colon	52	3.1500 (2.8712‐3.6368)	
Tumor size(mm)	＜30	41	3.0900 (2.3965‐3.6244)	.969
	≥30	58	3.0925 (2.7213‐3.5116)	
	Unknown	43		
Lymph node metastasis	No	63	3.3800 (3.0855‐3.7980)	.078
	Yes	79	2.9600 (2.4983‐3.2955)	
Distant metastasis	No	108	3.3900 (3.0624‐3.6286)	**.019**
	Yes	34	2.4625 (1.7985‐3.1648)	

### 2.2 Isolation of exosomes

Isolation of exosomes was performed by ultracentrifugation as previously described.^14^ Briefly, the serum was centrifuged at 10 000 *g* at 4°C for 30 minutes. Then, the exosomes were separated by ultracentrifugation (Class H, R, and S Preparative Ultracentrifuges, Type 50.4 Ti Rotor; Beckman Coulter) at 100 000 *g* for 120 minutes at 4°C. Next, the exosome pellets were washed with 1 mL PBS and treated with Trizol (Van Allen Way) for RNA isolation.

### 2.3 Transmission Electron Microscopy (TEM)

The exosome pellets were transferred to the grids in a 50 µL drop of 1% glutaraldehyde for 5 minutes and then transferred to a 100 µL drop of distilled water. The grids were allowed to stand for 2 minutes. Then, the grids were placed directly to a 50 µL drop of uranyl‐oxalate solution, pH 7, for 5 minutes and a glass dish covered with parafilm on ice. The grids were washed seven times with distilled water for 2 minutes each and examined using a JEM‐1200EX transmission electron microscope (JEOL, Japan) operated at 100 kV.

### 2.4 qNano

Exosome size and particle concentration were analyzed with TRPS (qNano; Izon Science Ltd) according to the manufacturer's instructions. Particle concentration was standardized by calibration beads of 1.0 × 10^13^ particles/mL.^15^ Data were analyzed using the Izon Control Suite software v.3.3.2.2000 (Izon Science Ltd).

### 2.5 Western blot analysis

The protein extracts were separated using 12% SDS‐PAGE and transferred onto a PVDF membrane (Millipore). The membranes were blocked with 5% evaporated skimmed milk in TBS (50 mmol/L Tris‐HCl, pH 7.5, 150 mmol/L NaCl) containing 0.1% Tween‐20 for 2 hours and incubated overnight at 4°C with the appropriate primary Ab, followed by incubation with HRP‐coupled secondary Ab for 1 hour at room temperature. Furthermore, the protein bands were visualized on photographic film using ECL blotting detection reagents (P0018; Beyotime). The following primary antibodies were used for western blotting: anti‐CD63, anti‐GM130, and anti‐TSG101 (Proteintech, America).

### 2.6 miRNA profiling and RNA‐sequence data analysis

A total of 3 μg RNA from each sample was used as input material for generation of the small RNA library. After cluster generation, the libraries were sequenced on an Illumina Hiseq 2500/2000 platform, and 50‐bp single‐end reads were generated. After sequencing, the data were subjected to the following preliminary analyses, which were performed by the Novogene Corporation: quality control analysis, read mapping to the reference sequence, known miRNA alignment, source tag removal, novel miRNA prediction, small RNA annotation summarization, miRNA editing analysis, miRNA family analysis, target gene prediction, miRNA quantification, mRNA differential gene expression analysis, and KEGG enrichment analysis.

### 2.7 RNA isolation and Real‐time PCR

Total serum RNA was harvested with the TRIzol reagent (Van Allen Way) according to the manufacturer's instructions. The RNA was reverse‐transcribed to cDNA with the Mix‐X miRNA First‐Strand Synthesis Kit (Takara Bio) according to the manufacturer's instructions. Then, real‐time PCR was performed using TB‐Green Premix Ex Taq II reagent (Takara Bio) according to the manufacturer's instructions. Each sample was analyzed in duplicate. The PCR was evaluated by melting curve analysis. Quantitative PCR analysis was performed using the LC480 (Applied Biosystems). The relative expression of exosomal miR‐320d in serum samples was evaluated by ΔCT (Ct^miRNA^‐Ct^U6^) as previously described.^16^ U6 was used as an internal control.

### 2.8 Statistical analysis

SPSS 22.0 (IBM) software and GraphPad Prism 6.0 (GraphPad Software) were used for statistical analysis. The data were presented as the median with interquartile range. The data between two groups were compared using the Mann‐Whitney *U* test. Receiver operator characteristic curves with corresponding C statistics (area under the curve, AUC) based on logistic models were used to determine the corresponding cutoff points with the pathological diagnosis treated as the “gold standard.” *P* < .05 was considered to be statistically significant for all contrasts.

## 3 RESULTS

### 3.1 The identification of exosomes

**Figure 1:**
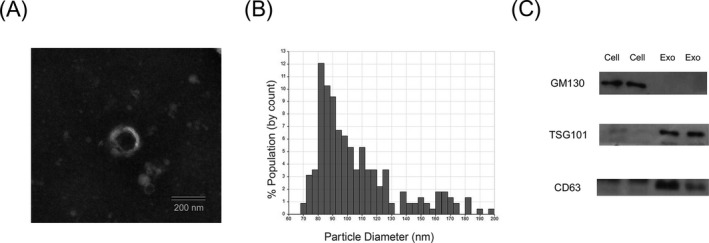
Exosome characterization and quantification. A, Transmission electron microscopy image showing representative data of exosomes of 50‐150 nm diameter from CRC patients [scale bar: 50 nm; high voltage (HV) = 80‐120 kV]. B, Distribution of exosomes of 50‐150 nm diameter in the samples from patients with metastatic CRC based on the qNano system (Izon Science Ltd). C, Western blot analysis showing exosome markers CD63 and TSG101 in the exosome‐enriched conditioned medium but not in the cell lysate. In contrast, cis‐Golgi matrix protein (GM130) was only observed in the cell lysate and not in the exosome fraction

Serum exosomes from CRC patients were isolated by ultracentrifugation and confirmed by TEM, qNano, and Western blot analysis. As shown in Figure 1A, TEM showed typical oval‐shaped extracellular vesicles of 50‐150 nm diameter. Moreover, the qNano system revealed the diameters of most exosomes concentrated on the 50‐150 nm (Figure 1B). In addition, a high level of CD63 and TSG101, the exosomal protein markers, was detected in exosomes but not in the whole cell extract, whereas 130 kDa GM130 (negative control) was only observed in the cell lysates but not in the isolated serum exosomes (Figure 1C).

### 3.2 Exosomal miRNA profile of CRC patients

**Figure 2:**
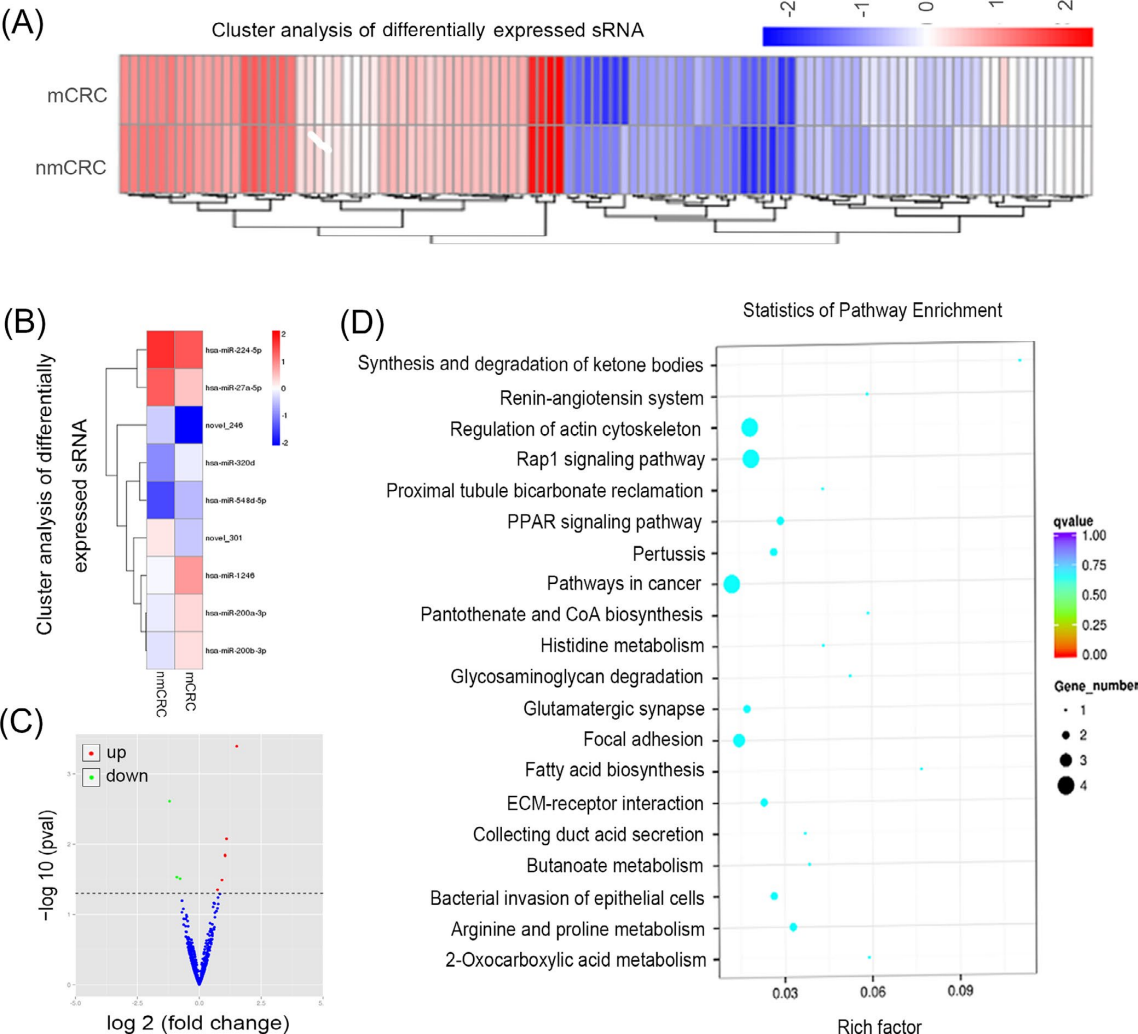
Exosome differential miRNA expression profile analysis. A, B, Cluster analysis of differentially expressed miRNAs. C, The expression profile changes of miRNAs in the volcano plot indicate up‐ and downregulated miRNAs in mCRC as compared to nmCRC. D, Candidate target gene Kyoto Encyclopedia of Genes and Genomes (KEGG) enrichment analysis

To identify the specific miRNA profile in the serum exosomes of the CRC patients, the RNA‐sequence analysis of samples from six CRC patients (three mCRCs and three non‐metastatic CRCs, nmCRCs) was examined. The raw miRNA expression profiling data included 1145 miRNAs, which could discriminate between metastatic and non‐metastatic CRC (Figure 2A). Meanwhile, the hierarchical clustering analysis revealed nine differentially expressed miRNAs (Figure 2B). Among these, three were downregulated and six were upregulated in the exosomes of CRC patients (Figure 2C), listed in Table 2. In addition, Kyoto Encyclopedia of Genes and Genomes (KEGG) analysis was performed to explore the significant pathways of the differentially expressed genes. We listed the 20 predicted signaling pathways of the selected miRNA (Figure 2D). Among these, pathways in cancer, Rap1 signaling pathway, regulation of actin cytoskeleton, focal adhesion, and PPAR signaling pathway seemed to be mainly involved in exosomal miRNA functions in CRC.

**Table 2:** Upregulated and downregulated miRNAs of CRC patients

miRNA	Fold change	*P*‐value	Description
hsa‐miR‐224‐5p	1.66630212	.04448	Up
hsa‐miR‐548d‐5p	1.8877399	.03229	Up
hsa‐miR‐200a‐3p	2.05793869	.01427	Up
hsa‐miR‐320d	2.06365242	.01467	Up
hsa‐miR‐200b‐3p	2.14622302	.00838	Up
hsa‐miR‐1246	2.85719599	.00041	Up
novel_246	−2.29803377	.00245	Down
novel_301	−1.87088386	.02936	Down
hsa‐miR‐27a‐5p	−1.70977917	.03098	Down

### 3.3 Exosomal miR‐320d as biomarker for CRC metastasis

**Figure 3:**
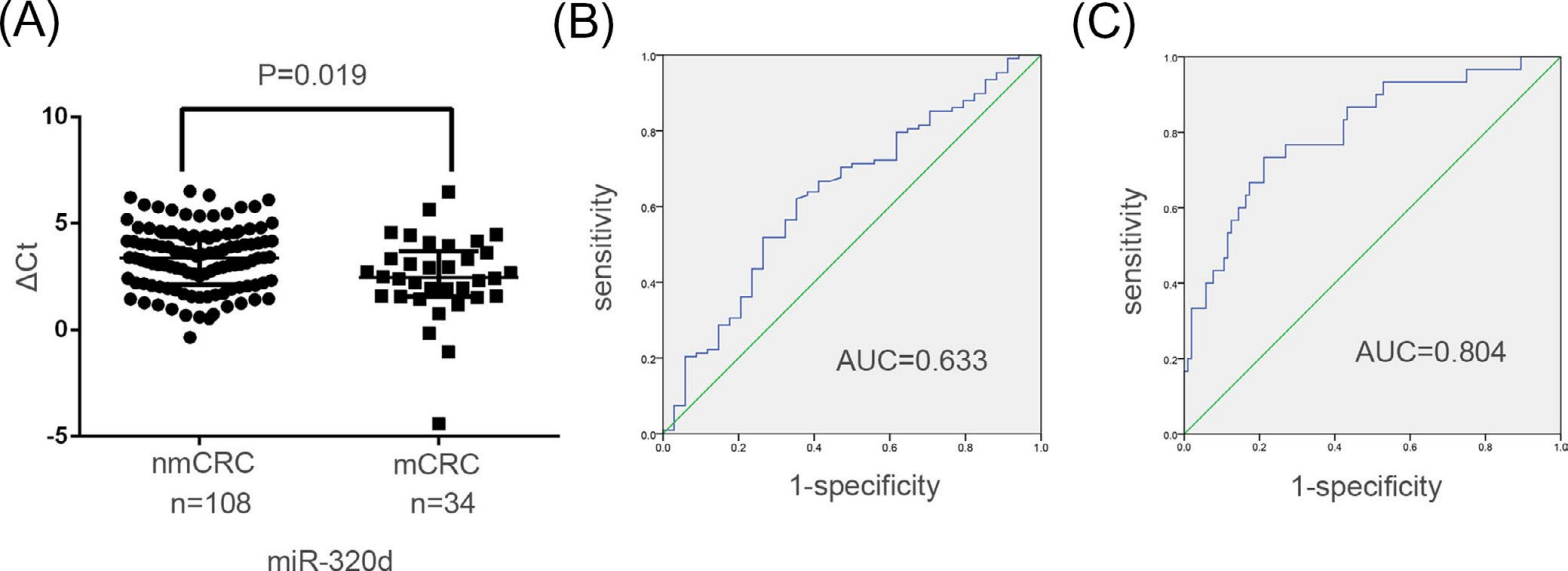
Exosomal miR‐320d level was significantly upregulated in mCRC patients. A, Mann‐Whitney *U* test indicated significant differences in miR‐320d levels between metastatic vs non‐metastatic CRC. Data are expressed as median with interquartile range (*P* = .019). B, The AUC of serum exosomal miR‐320d was 0.633 in 34 mCRC patients and 108 nmCRC patients. C, The combination of miR‐320d and CEA had an AUC of 0.804 with a sensitivity of 63.3% and specificity of 91.3%

We detected the selected miRNA expression levels by quantitative real‐time PCR. Nine differential exosomal miRNAs (novel_246, novel_301, hsa‐miR‐27a‐5p, hsa‐miR‐224‐5p, hsa‐miR‐548d‐5p, hsa‐miR‐200a‐3p, hsa‐miR‐320d, hsa‐miR‐200b‐3p, and hsa‐miR‐1246) were selected for large‐scale validation with independent serum samples from 142 CRC patients (34 mCRC patients and 108 nmCRC patients). We designed related primers and verified in cell and serum exosomes by qPCR. Due to low expression and low specificity of primers, five miRNAs were ruled out, and four were subjected to further verification in a large sample size. As shown in Figure 3A, only exosomal miR‐320d level was significantly upregulated in mCRC patients (*P* = .019), but not other three (Figure S1). The clinical characteristics of the 142 CRC patients are shown in Table 1. The serum expression levels of miR‐320d were not associated with age, gender, drinking status, and histological type.

To evaluate the diagnostic performance of miR‐320d for CRC metastasis, a receiver‐operating characteristic (ROC) curve was calculated. As shown in Figure 3B, AUC of exosomal miR‐320d was 0.633 (95% CI: 0.526‐0.740), with the sensitivity of 62.0% and the specificity of 64.7%. In addition, the combination of miR‐320d and CEA (carcinoembryonic antigen, CEA is a recommended prognostic marker in CRC for tumor diagnosis and monitoring response to therapy) had an AUC of 0.804 with a sensitivity of 63.3% and specificity of 91.3% (Figure 3C).

In summary, our data indicated that exosomal miR‐320d is a promising diagnostic biomarker for distinguishing metastatic from non‐metastatic CRC.

## 4 DISCUSSION

This study aimed to identify differential miRNAs in exosomes of CRC patients and to investigate the potential of exosomal miRNA as a biomarker for predicting CRC metastasis. Recent studies have suggested that exosomes could serve as cancer biomarkers since they carry several validated and surrogate non‐invasive biomarkers with diagnostic, prognostic, and predictive value.^17^

Given that we prospectively isolated and characterized the exosomes from the serum of mCRC or nmCRC patients by TEM, qNano, and Western blot, exosomal miR‐320d level could significantly distinguish mCRC patients from nmCRC patients, processing AUC values of 0.633 with the sensitivity of 62.0% and the specificity of 64.7%. We also assessed the diagnostics efficiency of the combination between miR‐320d and CEA, processing dramatical AUC values of 0.804 with a sensitivity of 63.3% and specificity of 91.3% to identify mCRC patients, thereby suggesting exosomal miR‐320d could be a valuable biomarker for mCRC diagnostics.

MiRNAs play important roles in oncogenesis and metastasis and could be used for diagnosis and prognosis of different types of cancers. Recently, miRNAs have shown significant promise as prognostic and diagnostic markers in CRC because of their unique expression profile in different diseases and the critical regulatory effects in carcinogenesis, tumor progression, invasion, angiogenesis, and metastases.^18^ There were many reports of aberrant miR‐320 expression in different cancers. miR‐320 expression was reported to be downregulated in breast cancer,^19^ glioma,^20^ and ovarian cancer.^21^ However, the expression level of has‐miR‐320 is highly related to the migration and invasion of ovarian cancer.^22^ The high expression of has‐miR‐320 was a direct indicator of negative prognosis and high risk of metastasis.^22^ MiR‐320b was found to be upregulated in CRC with liver metastasis and positively regulated the expression of metastasis promoting genes.^23^ MiR‐320d was found to be highly expressed in the proliferative compartment of the colonic crypts of normal colonic mucosa in CRC.^24^ Moreover, serum miR‐320a was found to be upregulated in stage IV CRC as compared to stage I‐II.^25^ These are consistent with our findings that the expression of exosomal miR‐320d was upregulated in mCRC.

Interestingly, studies have indicated that miR‐320 family members (miR‐320a, miR‐320b, miR‐320c, miR‐320d, and miR‐320e) were downregulated in interstitial cystitis (IC) tissues.^26^ In contrast to this study, a miRNA expression profile of IC generated by PCR‐based microarray analysis in a previous study showed that miR‐320 was upregulated in IC tissues.^27^ The instability of miRNAs may be responsible for these discrepancies among the studies.^9^ A recent study demonstrated that miRNAs are preserved in a stable form in the exosome, protected from endogenous RNase activity, and are important in cell‐to‐cell information processing.^4^, ^28^, ^29^, ^30^, ^31^, ^32^, ^33^ However, most studies that examined the potential of miRNAs as a biomarker did not use the exosomes. In this study, we used exosome samples separated from serum and examined their diagnostic value as biomarkers for mCRC patients.

As in most other malignant tumors, the metastasis of CRC is a very complicated process that involves multiple signal pathways and various mechanisms. In this study, KEGG pathway analysis indicated that the genes associated with the dysregulated miRNAs in the metastatic CRC group were mostly enriched in 20 KEGG pathways. Among these, signaling by Rap1/Rac1 is one of the major pathways controlling cancer cell migration and tumor metastasis.^34^ Besides, pathways in cancer also play a critical role in CRC metastasis.^35^ However, more research is warranted to elucidate the precise molecular mechanisms of exosomal miRNAs in mCRC.

This study had several limitations. First, long‐term clinical follow‐up data of each CRC patient were absent, which currently limit the ability to explore the prognostic value of miR‐320d. Second, the results of this small sample size preliminary study require further confirmation in large prospective studies.

In summary, this study clearly demonstrated that miR‐320d could be a useful blood‐based biomarker for distinguishing metastatic from non‐metastatic CRC. This non‐invasive biomarker may have great potential to predict the clinical behavior of CRC and monitor tumor metastasis.

## CONFLICT OF INTEREST

There are no conflicts of interest.
