# Supplementary figures and images for: Tumor‐derived exosomal miRNA‐320d as a biomarker for metastatic colorectal cancer
Source: J Clin Lab Anal. 2019 Aug 16;33(9):e23004. doi: 10.1002/jcla.23004 (PMC6868417; doi:10.1002/jcla.23004)

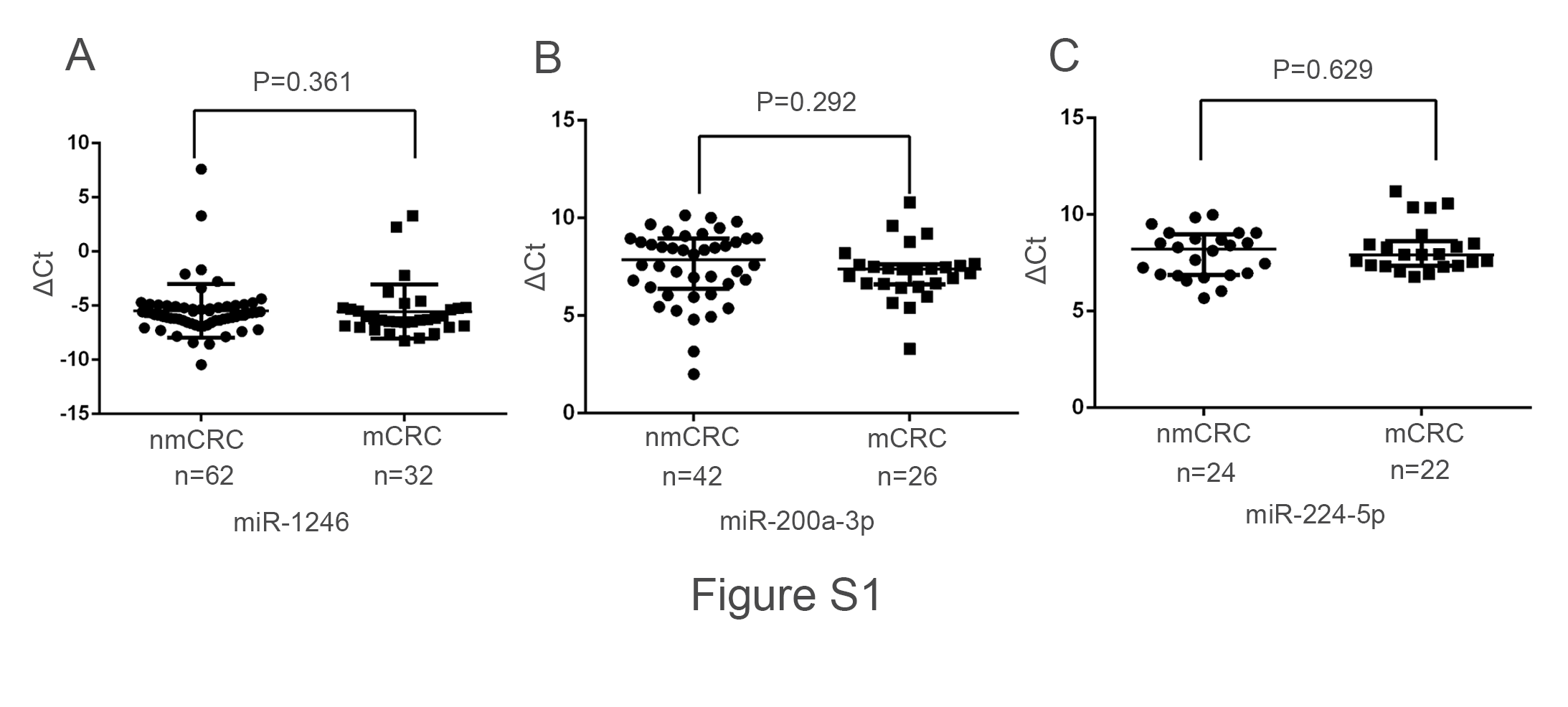

Supplement: Supplementary file 1 [file JCLA-33-na-s001.tif]
